# Antiproliferation of Berberine in Combination with Fluconazole from the Perspectives of Reactive Oxygen Species, Ergosterol and Drug Efflux in a Fluconazole-Resistant *Candida tropicalis* Isolate

**DOI:** 10.3389/fmicb.2016.01516

**Published:** 2016-09-23

**Authors:** Jing Shao, GaoXiang Shi, TianMing Wang, DaQiang Wu, ChangZhong Wang

**Affiliations:** ^1^Laboratory of Microbiology and Immunology, School of Chinese and Western Integrative Medicine, Anhui University of Chinese MedicineHefei, China; ^2^Laboratory of Biochemistry and Molecular Biology, School of Chinese and Western Integrative Medicine, Anhui University of Chinese MedicineHefei, China

**Keywords:** synergy, berberine, *Candida tropicalis*, resistance, efflux pump, ergosterol

## Abstract

*Candida tropicalis* has emerged as an important pathogenic fungus in nosocomial infections due to its recalcitrant resistance to conventional antifungal agents, especially to fluconazole (FLC). Berberine (BBR) is a bioactive herbal-originated alkaloids and has been reported to possess antifungal functions against *C. albicans*. In this paper, we tried to figure out the antifungal mechanisms of BBR and/or FLC in a clinical *C. tropicalis* isolate 2006. In the microdilution test, the minimum inhibitory concentration (MIC) of BBR was found 16 μg/mL with fractional inhibitory concentration index (FICI) 0.13 in *C. tropicalis* 2006. The synergism of BBR and FLC was also confirmed microscopically. After the treatments of BBR and/or FLC, the studies revealed that (i) FLC facilitated BBR to increase reactive oxygen species (ROS), (ii) FLC enhanced the intranuclear accumulation of BBR, (iii) BBR decreased the extracellular rhodamine 123 (Rh123) via inhibiting efflux transporters, (iv) FLC assisted BBR to reduce ergosterol content, and (v) BBR in combined with FLC largely downregulated the expressions of Candida drug resistance 1 (*CDR1*) and *CDR2* but impact slightly multidrug resistance 1 (*MDR1*), and upregulate the expression of ergosterol 11 (*ERG11*). These results suggested that BBR could become a potent antifungal drug to strengthen FLC efficacy in FLC-resistant *C. tropicalis* via ROS increase, intracellular BBR accumulation, ergosterol decrease and efflux inhibition.

## Introduction

The increased incidence of invasive fungal infection caused by *Candida* species (accounting for approximately 80%) is of the main responsibility for high morbidity and mortality in hospitalized patients (Weinstein et al., 2001; Snydman, 2003; Lass-Flörl, 2009). Although *Candida albicans* remains the most common pathogenic fungi encountered, the frequency of isolation of non-*Candida albicans Candida* (NCAC) species, i.e., *C. tropicalis*, *C. krusei*, *C. glabrata* and *C. parapsilosis*, has increased. In recent decades, *C. tropicalis* has emerged as the first or second NACA species in patients with cancer, neutropenia, malignancy and long-term medication, and was frequently isolated from bloodstream (candidemia) and urinary tract (candiduria) infections (Negri et al., 2012; Jiang et al., 2013). *C. tropicalis* does not only have high proportions in India, Brazil, and Taiwan, but is also becoming a critical public health problem in China (Colombo et al., 2007; Ruan and Hsueh, 2009; Jiang et al., 2013).

The rising *C. tropicalis* infections can be mainly attributed to the resistance to the conventional antifungal agents, such as azoles especially (Negri et al., 2012). Fluconazole (FLC), one of the first-line antifungal agents, is the most common used azoles for prophylaxis and therapy to combat candidemia in clinical practice. The clarified target of FLC is the lanosterol 14-α demethylase, a key enzyme responsible for the synthesis of ergosterol which is a pivotal component in candidal cell membrane encoded by *ERG11* (Zavrel and White, 2015). Except the mutation and overexpression of *ERG11*, respiration deficiency leading to decreased reactive oxygen species (ROS) and upregulation of drug efflux pump mediated by Cdr1p, Cdr2p belonging to ATP-binding cassette superfamily (APC transporter) and Mdr1p, a member of major facilitator superfamily (MFS) have also been recently documented to result in antifungal resistance of *C. tropicalis* to FLC (Vandeputte et al., 2005; da Silva et al., 2013, 2014; Forastiero et al., 2013; Jiang et al., 2013). The ever-increasing threat to FLC resistance calls for an urgent search for novel antifungal agents which possess strong antifungal activities alone or/and improve antimycotic potential of FLC in *C. tropicalis* clinical isolates.

Berberine (BBR), a bioactive herbal-originated alkaloid, has been traditionally used in the treatments of gastroenteritis, diarrhea, cholera, liver disease, inflammations and cancers due to its anti-diarrhoeal, anti-malarial, anti-secretory and anti-inflammation as well as anti-cancer effects with relatively low cytotoxicity *in vivo* and *in vitro* (Chung et al., 1999; Zeng et al., 2003; Kuo et al., 2004; Mantena et al., 2006). Recent studies have been also demonstrated the favorable antibacterial and anti-candidal activities of BBR against *Staphylococcus epidermidis*, *Staphylococcus aureus*, *Escherichia coli*, *C. albicans*, *C. krusei*, *C. glabrata*, and *C. parapsilosis* alone and/or in combination with FLC under planktonic and/or biofilm conditions (Yu et al., 2005; Wang et al., 2009; Iwazaki et al., 2010; Wei et al., 2011; Kong et al., 2012; Li et al., 2013). However, the antifungal effect of BBR on FLC-resistant *C. tropicalis* and the underlying mode of action remain largely unknown.

In the present study, we investigated the susceptibility of BBR alone and in concomitant use with FLC to a FLC-resistant *C. tropicalis* isolate via broth microdilution method, checkerboard assay and time-kill test. The morphology changes were inspected by scanning electron microscope (Vishnubalaji et al., 2016) and fluorescent microscope. The measurements of reactive oxygen species (ROS) and intracellular BBR accumulation were performed through DAPI and DHR-123 staining. The efflux capability and ergosterol content were surveyed by Rh 123 assay and HPLC method. Additionally, the critical genes related to efflux pump and ergosterol synthesis, i.e., *CDR1*, *CDR2*, *MDR1* and *ERG11*, were analyzed by quantitative reverse transcription polymerase chain reaction (qRT-PCR).

## Materials and Methods

### Isolates and Cultivation

*Candida tropicalis* ATCC750 was procured from NanJing Bian Zhen Biotechnology Co., LTD. (Jiangsu, China), and the clinical *C. tropicalis* isolate 2006 used in this study was kindly provided by Clinical laboratory of Anhui Provincial Hospital (Anhui, China). *C. parapsilosis* ATCC 22019 as the quality control was kindly provided by Prof. YuanYing Jiang from College of Pharmacy, Second Military Medical University (Shanghai, China). All isolates were stored in YPD medium (1% yeast extract, 2% peptone, 2% dextrose) and 20% glycerol at -80°C. The isolates were subcultured on Sabouraud Dextrose Broth (SDB) for 24 h at 37°C, and then the cells were harvested by 7500 rpm centrifugation and washed twice by sterile phosphate-buffered saline (PBS). The fungal cells were resuspended in RPMI-1640 medium (Invitrogen, Carlsbad, CA, USA) and cell density was calculated using a hemocytometer for use.

### *In vitro* Antifungal Activity

**FIGURE 1:**
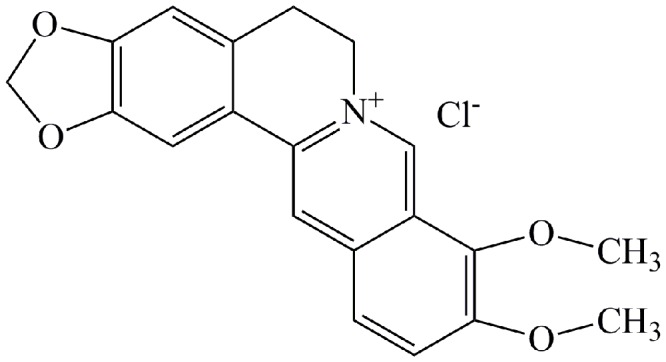
**Molecular structure of berberine (BBR)**.

Microdilution method was adopted to determine the minimal inhibitory concentration (MIC) of berberine (Xiao Cao, Xi’an, China, **Figure 1**) and FLC (National Institutes for Food and Drug Control, Beijing, China). Briefly, the cell suspension (100 μL, 2 × 10^3^ CFU/mL) was added into 96-well polystyrene microtiter. After 1-h adhesion phase at 37°C, the non-adherent cells were washed away by PBS. The concentrations of BBR were serially twofold diluted in a range from 1 to 512 μg/mL, and those of FLC were prepared ranging from 0.125 to 1024 μg/mL. After 24-h incubation at 37°C, the MIC_80_ was defined as the lowest concentration of the agent at which there was 80% inhibition of visible growth compared with the control at the wavelength of 630 nm by a spectrophotometer (SpectraMax M2/M2e, Wang Lab Sunnyvale, Stanford, CA, USA). Subsequently, the checkerboard technique was performed to evaluate the synergism of BBR and FLC. Briefly, the fungal cells were diluted to 2 × 10^3^ CFU/mL in RPMI-1640 medium and added into each well. Then the cells were exposed to various concentrations of FLC (0.03125-32 μg/mL) in combination with different concentrations of BBR (0.25–16 μg/mL). The interaction between BBR and FLC was determined by calculating the fractional inhibitory concentration index (FICI) as follows: FICI = (MIC_BBR_ in combination/MIC_BBR_ alone) + (MIC_FLC_ in combination/MIC_FLC_ alone), in which synergism was interpreted as FICI ≤ 0.5, indifference was defined as 0.5 < FICI ≤ 4.0, and antagonism was FICI > 4.0.

### Time-Kill Assay

The exponentially growing *C. tropicalis* 2006 cells were harvested, resuspended in fresh RPMI 1640 medium, and adjusted to 5 × 10^4^ CFU/mL. The concentrations of BBR and FLC were set at 2 and 8 μg/mL according to each MIC in combination, respectively. The cells were grown at 37°C with constant shaking. At designated time points (0, 2, 4, 6, 8, 12, 24, and 48 h), the cell broth was pipetted out, centrifugated and rinsed by sterile PBS before they were counted on sabouraud dextrose agar (Dimaras et al., 2012). The control was free of FLC and BBR. Compared to the most active drug alone, an increase/decrease in killing of ≥2log_10_CFU/mL for the combination was defined as synergy/antagonism, while the killing effect for the combination of within 2log_10_CFU/mL compared with either agent alone was defined as indifference.

### Scanning Electron Microscope

The fungal supernatant (=2 mL, 2 × 10^6^ CFU/mL) containing agents (2 μg/mL BBR, 8 μg/mL FLC, and 2 μg/mL BBR plus 8 μg/mL FLC) were loaded on 6-well plate containing sterile coverslips and incubated for 24 h at 37°C. The control had medium with no drugs. After incubation, the coverslip was taken out and rinsed by sterile PBS. The samples were initially fixed in 2.5% glutaraldehyde overnight at 4°C, and post-fixed with 0.1% osmium tetroxide for 1 h. Then, the samples were dehydrated sequentially by 30, 50, 70, 90, 95, and 100% ethanol for each step 20 min. After critical point drying, the coverslips were sputter-coated with gold in a vacuum evaporator for morphological observation by SEM JSM-6700F (JEOL, Musashino-shi, Tokyo, Japan).

### Fluorescein Diacetate (FDA) Assay

The fungal broth was adjusted to 2 × 10^6^ CFU/mL and incubated respectively with 2 μg/mL BBR, 8 μg/mL FLC, and 2 μg/mL BBR plus 8 μg/mL FLC samples for 24 h at 37°C. The control was free of drugs. The stock solution of fluorescein diacetate (FDA, Sigma, Shanghai, China) was dissolved by acetone to a concentration of 10 mg/mL and stored at -20°C prior to use. A 1:50 working solution of FDA was freshly diluted in sterile PBS before each assay. Subsequently, 100 μL working solution of FDA plus 100 μL fungal solution was added into each pre-rinsed well in a 96-well microtiter plate. The plate was incubated in the dark at 37°C for 30 min on a rocking table. The fluorescence in the well were recorded with a fluorescent microscope (Olympus IX81, Tokyo, Japan) at the excitation wavelength of 494 nm and the emission wavelength of 518 nm, respectively (Peeters et al., 2008).

### Measurement of Intracellular Reactive Oxygen Species

The endogenous ROS in *C. tropicalis* 2006 cells was measured with the fluorescent dye dihydrorhodamine-123 (DHR-123, Sigma–Aldrich, USA) as described previously (Hao et al., 2013). Briefly, the cells (1 mL, 2 × 10^6^ CFU/mL) were suspended in RPMI-1640 medium and mixed with different concentrations of BBR and/or FLC for 24 h at 37°C. After incubation, the cells were harvested via centrifugation at 7500 rpm for 5 min, washed with sterile PBS, resuspended in sterile PBS (6 × 10^6^ CFU/mL), and stained with 5 μg/mL DHR-123 in the dark at 37°C for 30-min incubation. The results were recorded and analyzed by a FACSCalibur flow cytometer (Becton-Dickinson, USA).

### Intracellular BBR Accumulation Assay

Intracellular BBR concentration was detected according to a previously described protocol with a few modifications (Li et al., 2013). Briefly, the isolate broth (=1 mL, 2 × 10^6^ CFU/mL) containing BBR (=2, 4, and 8 μg/mL) was cultured at 30°C for 0, 2, 4, 6, and 8 h. After 3000 *g* of centrifugation and twice washings, the pellets were resuspended in 1 mL of PBS. Then, 100 ml of each sample was transferred into a flat-bottomed 96-well microplate (Greiner, Germany). The fluorescence of BBR was measured by a spectrophotometer (SpectraMax M2/M2e, Wang Lab Sunnyvale, Stanford, CA, USA) at the wavelengths of 405 nm excitation and 520 nm emission. The intracellular localization of BBR was performed according to the method as described previously (de la Fuente-Salcido et al., 2007). Briefly, after the exposure of fungal cells (=1 mL, 2 × 10^6^ CFU/mL) to BBR and/or FLC for 8 h, the pullet was harvested, washed, and treated with 1 μg/mL of 4′,6-diamidino-2-phenylindole (DAPI) at 30°C for 10 min in the dark. Living cells were immobilized on 0.1% poly-L-lysine-coated slides and observed with a fluorescent microscope (Olympus IX81, Tokyo, Japan) at the wavelengths of 340 nm excitation and 488 nm emission.

### Efflux Assay by RH123

The Rh123 (Sigma–Aldrich, Shanghai, China) assay was administered according to the previous procedures with minor modifications (Jiang et al., 2013). The fungal cells (=5 × 10^7^ CFU/mL) were collected and transferred to fresh RPMI 1640 for 4-h incubation at 35°C. Then the cells were harvested, washed and resuspended in sterile PBS buffer. BBR (= 2, 4, and 8 μg/mL), glucose (5%) and Rh123 (10 μM) were added to the cell suspension and incubated at 35°C in a shaker. After incubation for 0, 10, 20, 30, 40, 50, and 60 min, the samples were centrifuged at 7500 rpm for 5 min to pool the supernatants, and the fluorescence was measured by a spectrophotometer at the wavelengths of 501 nm excitation and 529 nm emission.

### HPLC Evaluation

At first, the sterol was extracted in accordance to the methods described before with a few modifications (Arthington-Skaggs et al., 1999). After the treatments of BBR (= 2 μg/mL) and/or FLC (= 8 μg/mL), the fungal cells were harvested by centrifugation at 7500 rpm for 5 min and washed twice with sterile PBS. The wet pellet was adjusted to 0.50 g, mixed with 2.5 mL of 15% alcoholic sodium hydroxide solution (15 g NaOH and 10 mL sterile distilled water, brought to 100 mL with 100% ethanol) and 1 mL sterile PBS, and agitated rigorously for 1 min. After incubation in a water bath at 90°C for 2 h, the cell suspensions were removed to cool at room temperature, and subsequently added with 3 mL of petroleum ether (Sinoreagent, China), followed by a vigorous vortex for 3 min. The organic phase was then transferred to a clean glass tube to evaporate petroleum ether in a water bath at 60°C. The extracted sterols were redissolved in 1 mL of methanol (Sinoreagent, China) prior to HPLC-UV analysis. The ergosterol content was detected using a modified HPLC (Agilent 1100LC, Shanghai, China) based on the method established previously (Peacock and Goosey, 1989). In brief, after the filtration (pore size 0.22 mm, Millipore, Shanghai, China), the sample containing ergosterol was analyzed at 282 nm with C18 reverse-phase column (ZorBax Eclipse Plus, 5, 4.6, and 100 mm, Agilent, USA). The mobile phase was a solution of methanol in dichloromethane (= 0.025%, v/v) at a flow rate of 1 mL/min with a race time of about 20 min.

### qRT-PCR Analysis

One milliliter cells (= 2 × 10^6^ CFU/mL) was transferred into a sterilized 6-well polystyrene microtiter plate (Corning, NY, USA). After incubation with BBR and/or FLC at 37°C for 24 h, the cells were harvested by 7500 rpm centrifugation for 5 min. The drug-free well was set as control. Pellets were washed thrice with sterile PBS, and total RNA was extracted by using MagExtractor-RNA kit (ToyoBo, Tokyo, Japan). cDNA was obtained through reverse transcription reaction using the ReverTra Ace qPCR RT Master Mix with gDNA Remover Kit (ToyoBo, Tokyo, Japan). The prepared cDNA was diluted 10 × fold before use. All experiments were performed on ice. Primers of *ERG11* (ergosterol 11), *CDR1* (Candida drug resistance 1), *CDR2*, *MDR1* (multidrug resistance 1) and *ACT1* (actin 1), (**Table 1**) were synthesized by Sangon Biotech (Shanghai, China). Real time PCR reaction was conducted with SYBR Green I. The reaction was run on ABI7000 fluorescent quantitative PCR system (Applied Biosystem, USA) with thermal cycling as follows: initial step at 95°C for 60 s, followed by 40 cycles at 95°C for 15 s, 55°C for 15 s, 72°C for 45 s. All data were normalized to housekeeping gene *ACT1*, the internal reference gene. Relative fold changes of the gene were calculated using the formula 2^-ΔΔCt^ (Xie et al., 2011).

**Table 1:** Primers used for RT-PCR.

Genes	Forward (5′-3′)	Reverse (5′-3′)
*ERG11*	CTACTCCCAAAAAAAACCATA	TAAACCTAATCCCAAGACATC
*CDR1*	TGGAAAGAGTTGGAGGGTATGTTA	TCCCAAGGTTTCGCCATC
*CDR2*	GCTTAGATGCCGCCACTG	AGCCCATTCTGATGAAATACTC
*MDR1*	TTGGCGTTAGAGGATTTACTTTGG	GAATGAAAACTTCTGGGAAAACTGG
*ACT1*	GACCGAAGCTCCAATGAATC	AATTGGGACAACGTGGGTAA

### Statistical Analysis

All experiments were performed in triplicate. The results were reported as mean ± standard deviation, calculated by *SPSS* 17.0, and analyzed using one-way ANOVA. The comparison among groups adopted *t*-test. *P* < 0.05 was considered as statistically significant.

## Results

### *In vitro* Antifungal Activities

**FIGURE 2:**
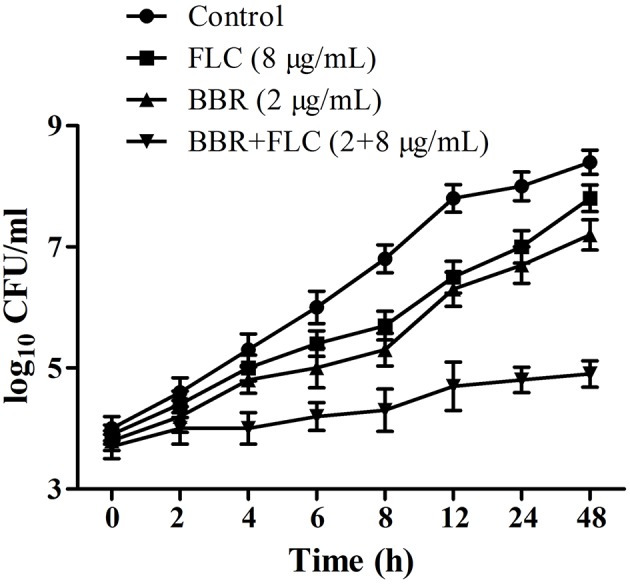
**Time-kill curves for *C. tropicalis* 2006 treated with BBR, FLC and BBR + FLC**.

**FIGURE 3:**
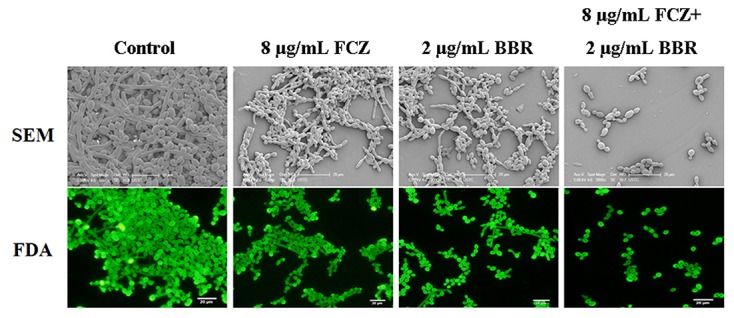
**The morphology of *C. tropicalis* 2006 treated with BBR, FLC and BBR + FLC by SEM (up, bar: 20 μm) and fluorescence microscopy (down, bar: 20 μm)**.

The antifungal activities of BBR and/or FLC were firstly assessed by broth microdilution method and checkerboard technique in *C. parapsilosis* ATCC22019, *C. tropicalis* ATCC 750 and *C. tropicalis* 03. The MIC_80_ of BBR alone were of 16 μg/ml, but reduced to 8 μg/ml in *C. parapsilosis* ATCC22019 and *C. tropicalis* ATCC 750, and 4 μg/ml in *C. tropicalis* 2006 when BBR was in combined use with FLC. Without BBR, the MIC_80_ of FLC were of 1024 μg/ml, and sharply decreased 128 fold to 8 μg/ml in *C. tropicalis* 2006 with the aid of BBR. Although the combined effect of BBR and FLC seemed indifference to *C. parapsilosis* ATCC22019 and *C. tropicalis* ATCC 750 (FICI = 1 and 0.75 > 0.5), both agents had synergistic effect on *C. tropicalis* 2006 with FICI 0.13 (**Table 2**). Subsequently, the time-kill test was used for further evaluating the synergism of BBR and FLC in *C. tropicalis* 2006. It could be observed that the antifungal activity of 8 μg/mL of FLC was inferior to that of 2 μg/mL of BBR during the tested time span. The required MIC of FLC decreased by the addition of BBR. At 48-h incubation, the two drugs could make an increase of 2.32 log_10_CFU/mL in killing compared to 2 μg/mL of BBR alone (the most active agent, **Figure 2**), indicating the synergism of 2 μg/ml of BBR and 8 μg/ml of FLC against *C. tropicalis* 2006. We also employed SEM and fluorescent microscope to survey the morphological changes of *C. tropicalis* 2006 after the exposures of BBR and/or FLC. Compared with the control, the hyphal cells were almost eradicated and only yeast-form cells remained when BBR and FLC were used concomitantly (**Figure 3**).

**Table 2:** Susceptibilities of BBR and/or FLC to *Candida tropicalis* isolates.

Isolates	MIC_80_ alone (μg/mL)		MIC_80_ in combination (μg/mL)		FICI	Interpretation
	BBR^a^	FLC^b^	BBR	FLC
*C. parapsilosis* ATCC 22019	16	0.5	8	0.25	1	IND ^c^
*C. tropicalis* ATCC 750	16	0.5	8	0.125	0.75	IND
*C. tropicalis* 2006	16	1024	2	8	0.13	SYN^d^

*^a^BBR, berberine; ^b^FLC, fluconazole; ^c^IND, indifference. ^d^SYN, synergism.*

### Intracellular ROS Accumulation

**FIGURE 4:**
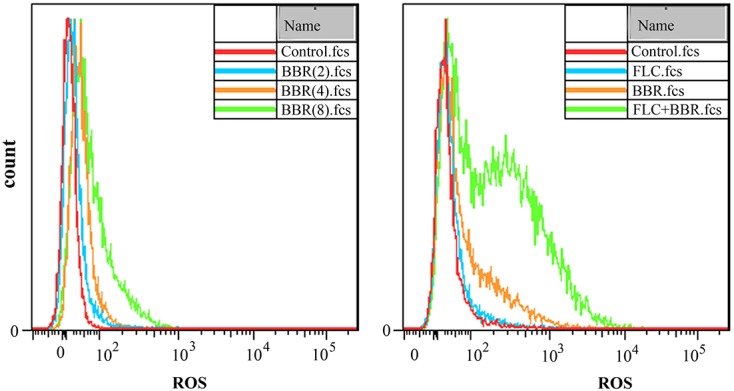
**Reactive oxygen species (ROS) analysis by flow cytometer after the exposures to BBR (Left) and BBR + FLC (Right) in *C. tropicalis* 2006**.

The ROS-specific dye DHR-123 could be oxidized to the fluorescent rhodamine 123 by the intracellular ROS that could be detected by fluorescent microscope. As shown, the intracellular ROS levels increased along with the BBR concentrations from 2 to 8 μg/ml. It could also be observed that the intracellular ROS level grew after the exposure to 2 μg/ml BBR + 8 μg/ml FLC more than those after the exposure to 2 μg/ml BBR or 8 μg/ml FLC in *C. tropicalis* 2006 (**Figure 4**).

### Intracellular BBR Accumulation

**FIGURE 5:**
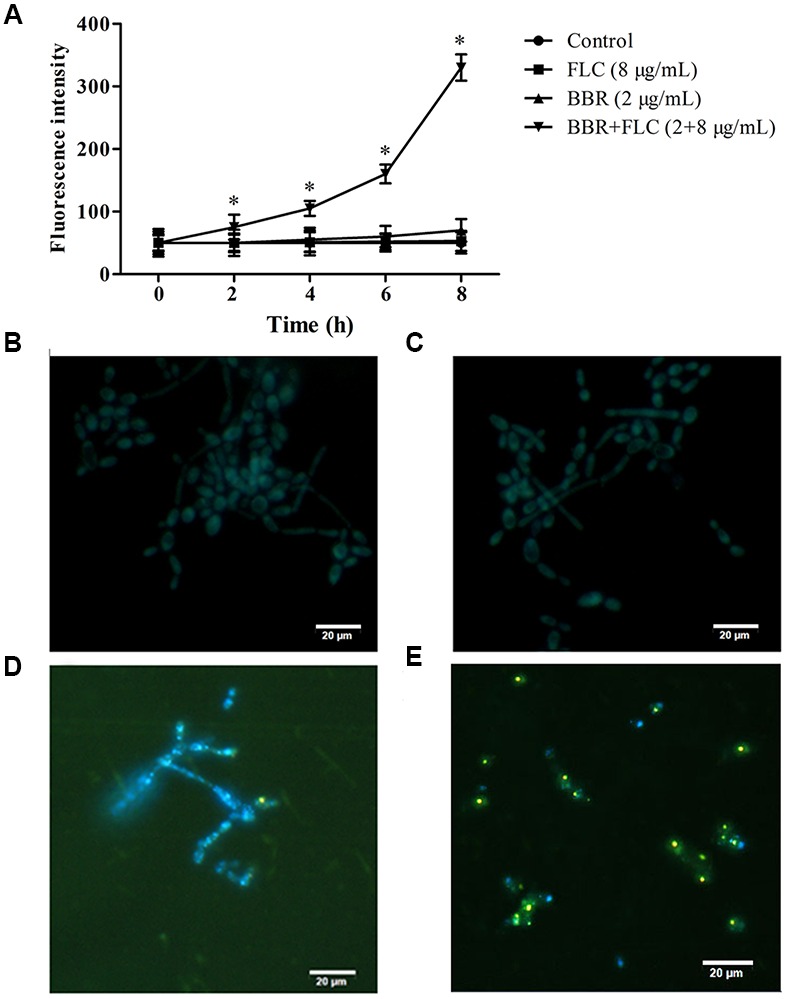
**(A)** The fluorescence intensities of BBR were measured by spectrophotometer at the wavelengths of 405 nm excitation and 520 nm emission in *C. tropicalis* 2006. The intracellular localization of BBR was observed by fluorescent microscope at the wavelengths of 340 nm excitation and 488 nm emission after the medications of **(B)** no drug (control), **(C)** FLC, **(D)** BBR, and **(E)** BBR + FLC in *C. tropicalis* 2006. ^∗^*p* < 0.05, compared with the control.

Compared with the control, the fluorescent intensities did not changed with significant differences in the BBR-treated (= 2 μg/ml) or FLC-treated (= 8 μg/ml) cells, but rose remarkably after 2 h of exposures to BBR and FLC in *C. tropicalis* 2006 (*p* < 0.05, **Figure 5A**). For further validation, we used fluorescent microscope to examine the intracellular localization of BBR which could emit yellow fluorescence (Li et al., 2013). Compared with the control, the FLC-treated cells had no fluorescence (**Figures 5B,C**), whereas there were BBR-emitted yellow fluorescence in BBR-treated cells (**Figure 5D**). After the exposure to BBR in combination with FLC, the visibly yellow fluorescence became strong (**Figure 5E**). Notably, the fluorescence emitted by BBR was particularly strong in the nucleus and overlapped with the nucleus dye DAPI (**Figure 5E**), suggesting that FLC could facilitate BBR to accumulate in *C. tropicalis* nucleus.

### Effect of BBR on RH123 Efflux

**FIGURE 6:**
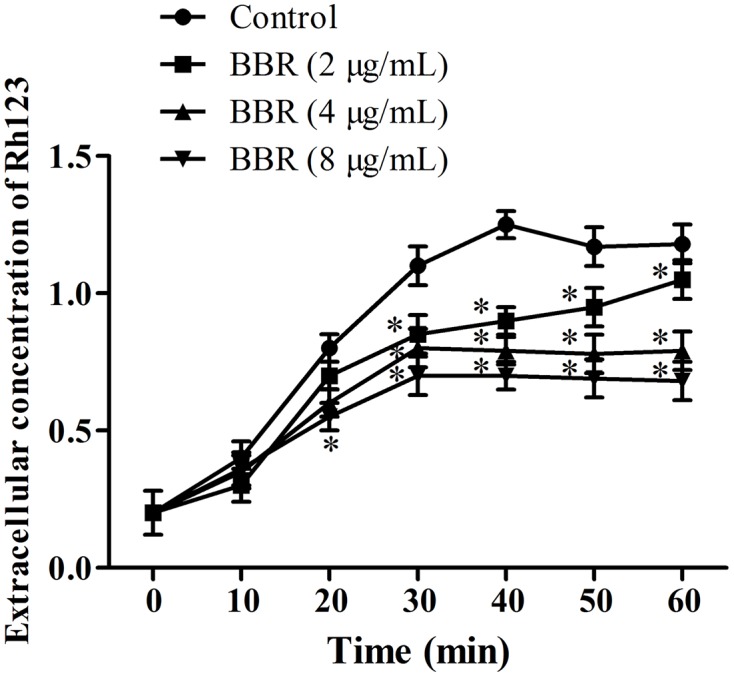
**The extracellular concentrations of Rh123 were detected by spectrophotometer at the wavelengths of 501 nm excitation and 529 nm emission after the co-incubations with BBR for 0, 10, 20, 30, 40, 50, and 60 min in *C. tropicalis* 2006.** ^∗^*p* < 0.05, compared with the control.

To clarify the relationship of intracellular BBR accumulation with efflux pump, we chose Rh123, a substrate of multidrug resistance (MDR) protein (Clark et al., 1996), to detect the extracellular Rh123 concentration. As shown, the extracellular Rh123 concentration increased along with the increasing BBR concentrations from 2 to 8 μg/ml. Compared with the control, the extracellular Rh123 concentrations rose significantly after 20 min of incubation with 8 μg/ml of BBR (*p* < 0.05) and 40 min of incubations with 2 and 4 μg/ml of BBR (*p* < 0.05, **Figure 6**).

### Ergosterol Analysis by HPLC

**FIGURE 7:**
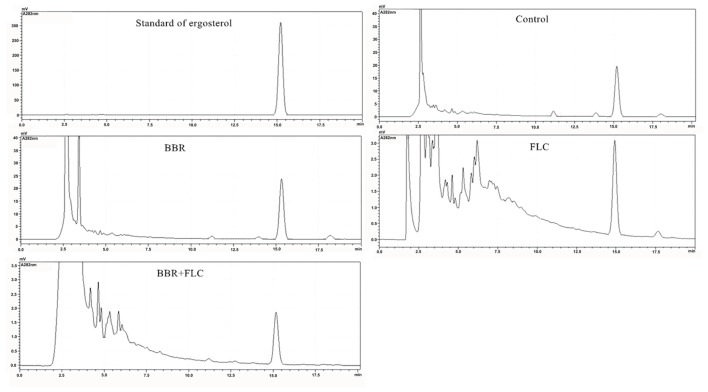
**Ergosterol content surveyed by HPLC after the administrations of BBR, FLC, and BBR + FLC in *C. tropicalis* 2006**.

The standard ergosterol peaked at about 15.26 min in HPLC (**Figure 7**). Compared with the control (**Figure 7**), the peak area seemed unchanged in the treatment of 2 μg/ml BBR (**Figure 7**), and declined by sevenfold when 8 μg/ml FLC was used (**Figure 7**). After the combined use of 2 μg/ml BBR and 8 μg/ml FLC, the peak area reduced by approximately twofold in comparison with that of 8 μg/ml FLC and 14-fold compared with the control (**Figure 7**).

### Gene Expressions by qRT-PCR

**FIGURE 8:**
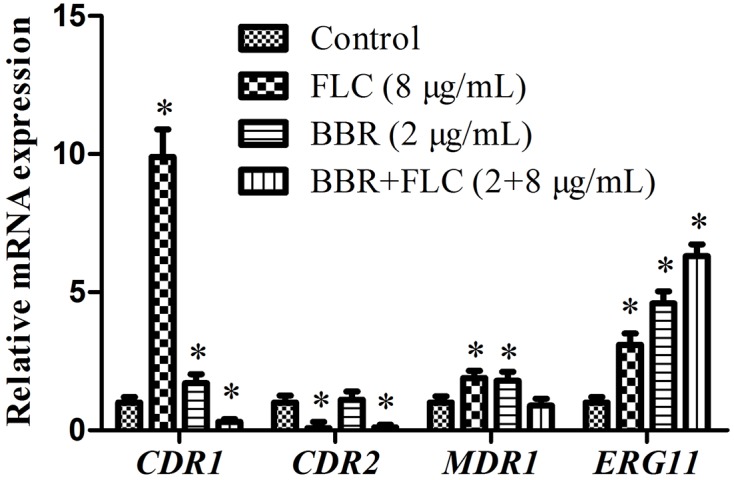
**Gene expressions of *CDR1*, *CDR2*, *MDR1* and *ERG11* in *C. tropicalis* 2006 under treatments**. ^∗^*p* < 0.05, compared with the control.

To further explain the possible involvements of efflux pump and ergosterol synthesis in the antifungal activity of BBR and/or FLC against FLC-resistant *C. tropicalis* 2006, the expressions of *CDR1*, *CDR2*, *MDR1* and *ERG11* genes were assessed by qRT-PCR. Compared with the control, it was observed that the expression of (i) *CDR1* increased 1.7-fold (*p* < 0.05), 9.9-fold (*p* < 0.05) and decreased 3-fold (*p* < 0.05), (ii) *CDR2* increased 1.1-fold and decreased 12.5-fold (*p* < 0.05), 10-fold (*p* < 0.05), (iii) *MDR1* increased 1.8-fold (*p* < 0.05) and 1.9-fold (*p* < 0.05), and decreased 1.1-fold, and surprisingly, (iv) *ERG11* increased 4.6-fold (*p* < 0.05), 3.1-fold (*p* < 0.05) and 6.3-fold (*p* < 0.05) when the exposed concentrations of BBR, FLC and BBR+FLC were of respectively 2, 8 and 2 + 8 μg/ml (**Figure 8**).

## Discussion

With widely use of intrusive catheters and extensively abuse of conventional antifungal agents, the fungal infections caused by C. tropicalis have become a serious health problem worldwide. The ever-increasing isolations of C. tropicalis resistant to FLC contribute to therapeutic failures. Currently, there are at least two common efforts for antifungal drugs to control the infections by C. tropicalis. One is to find or synthesize novel classes of potent antifungal agents with low toxicity, the other is to discover “old drugs” which have not been used for antifungal purpose before but can improve the antifungal activity of the conventional agents (such as FLC). The advancement of the former effort is quite slow, while the second effort becomes a promising alternative in the treatment of C. tropicalis. Meanwhile, it is meaningful to investigate the mechanism of action of the “old drugs” which will be helpful for understanding drug resistance and searching new drug targets (Vandeputte et al., 2005; Kothavade et al., 2010; da Silva et al., 2013; Neto et al., 2014; Choi et al., 2016).

Berberine existent in a variety of medicinal plants has been previously reported to possess broad-spectrum antimicrobial as well as antifungal activities. The published reports revealed that BBR seemed to be more potent against pathogenic fungi than bacteria. In *Candida* species, *C. tropicalis* appeared to be more susceptible to BBR than *C. albicans* (Wei et al., 2011). Several reports have demonstrated the synergism of BBR with FLC against *C. albicans*, but we did not find records on the antifungal activity of BBR combined with FLC in *C. tropicalis*. In this study, we demonstrated that BBR could improve the antifungal activity of FLC in a FLC-resistant *C. tropicalis* isolate 2006. To further define the synergism of the two drugs, however, we will employ more clinical *C. tropicalis* isolates resistant to FLC in a next experiment.

To clarify the antifungal mechanism of BBR in combination with FLC in *C. tropicalis* 2006, their individual antifungal mode of action should also acquire thoughtful considerations. In a previous study, the combination of BBR and FLC was reported to contribute to the augmentation of intracellular ROS accumulation via enhancing the tricarboxylic acid cycle and inhibiting the ATP-synthase activity in *C. albicans* (Xu et al., 2009). Our results showed that the ROS level increased alone with the rising of BBR, and BBR plus FLC could promote the endogenous ROS level in *C. tropicalis* 2006. Further experiments demonstrated that FLC could facilitate intracellular BBR accumulation and Rh123 outflow by hindering the normal function of Mdr1 which is an important efflux pump transporter belonging to the major facilitator superfamily (MFS). The accumulation of BBR promoted by FLC could enhance the antifungal activity of BBR in turn once BBR reached an effective intracellular concentration (Stermitz et al., 2000; Ball et al., 2006; Samosorn et al., 2009; Ettefagh et al., 2011). In addition, several lines of evidence indicated that BBR could be a DNA intercalator with preference to AT-rich sequence (Davidson et al., 1977; Iwazaki et al., 2010). Combined with fluorescence-emitting feature, we presumed that BBR could bind DNA and accumulate mainly in nucleus in *C. tropicalis* 2006.

Ergosterol is a major constitute of fungal plasma membrane and responsible for the integrity of cell membrane. The target of FLC is lanosterol 14α-demethylase which is pivotal in the synthesis of ergosterol (Zavrel and White, 2015). The changes of ergosterol content would deepen our understanding concerned with the combined mechanism of BBR and FLC. In this work, the ergosterol contents were remarkably reduced by FLC and BBR + FLC, inferring that FLC could assist BBR to inhibit the growth of *C. tropicalis* 2006 via interfering the integrity of cell membrane. These results were likely due to the fact that BBR could improve the permeability of cell membrane by inhibiting sterol 24-methyl transferase in the pathways of ergosterol (Park et al., 1999; Park et al., 2001; Kim et al., 2004). Interestingly, we noticed that BBR alone had no evident impact on ergosterol content. Accordingly, we assumed that the targets of BBR and FLC were different in cell membrane.

To further evaluate the effects of BBR and/or FLC on genes in charge of efflux transporter and ergosterol synthesis, the expressions of *CDR1*, *CDR2*, *MDR1* and *ERG11* genes were assessed by qRT-PCR. BBR or FLC alone was not fully effective to suppress the expressions of the former three genes, although our results indicated that BBR alone could accelerate the Rh123 outflow via inhibiting MDR1 protein. This inconsistency might be due to the fact that Rh123 and FLC did not, or at least partly, share a common MDR1-like transporter alike the situation in *C. albicans* (Clark et al., 1996). The relative significance among *CDR1*, *CDR2*, *MDR1* and *ERG11* have been well-documented and were different from that in *C. albicans*. Compared with *ERG11*, the efflux pump genes do not seem to play a crucial role in azole resistance in *C. tropicalis* (Vandeputte et al., 2005; Forastiero et al., 2013; Jiang et al., 2013). To our surprise, the expressions of *CDR1* and *CDR2* were sharply inhibited, while the expressions of *ERG11* appeared to be upregulated in the cases of BBR + FLC in this work. We hypothesized that on one hand the isolate used was limited in this work, and on the other hand, the targets of BBR and FLC might be controlled by *ERG11* although their targets were different as described above, and the pathogen had to ascend the expression of *ERG11* to compensate the losses caused by BBR and FLC but with no new synthesis of ergosterol. However, it is reasonable to take *ERG11* mutations including base-pair deletion and/or amino acid substitution into account in future studies.

In summary, we demonstrated here the synergism of BBR and FLC against FLC-resistant *C. tropicalis*, which is likely associated with intracellular BBR accumulation followed by endogenous ROS increase. In addition, we obtained a significant inhibition of ergosterol biosynthesis as well as a decrease of efflux transporter. The results also suggest that BBR can be a potential candidate for the treatment of resistant *C. tropicalis*.

## Author Contributions

JS processed the data and wrote the manuscript, GS performed the experiment, TW, DW, CW devised the experiment, CW examined the manuscript.

## Conflict of Interest Statement

The authors declare that the research was conducted in the absence of any commercial or financial relationships that could be construed as a potential conflict of interest.
